# Transcriptome analysis of adipose tissue in grazing cattle: Identifying key regulators of fat metabolism

**DOI:** 10.1515/biol-2022-0843

**Published:** 2024-04-25

**Authors:** Xia Qin, Xige He, Lu Chen, Yunfei Han, Yueying Yun, Jindi Wu, Lina Sha, Gerelt Borjigin

**Affiliations:** College of Food Science and Engineering, Inner Mongolia Agricultural University, #306 Zhaowuda Road, Saihan District, Huhhot, Inner Mongolia 010018, China; Pharmacy and Materials School, Huainan Union University, Huainan 232038, China

**Keywords:** adipose tissue, grazing cattle, house breeding cattle, microRNA, transcriptome analysis

## Abstract

The taste and tenderness of meat are the main determinants of carcass quality in many countries. This study aimed to discuss the mechanisms of intramuscular fat deposition in grazing and house-breeding cattle. We performed transcriptome analysis to characterize messenger RNA and microRNA (miRNA) expression profiles. A total of 456 and 66 differentially expressed genes (DEGs) and differentially expressed (DE) miRNAs were identified in the adipose tissue of grazing and house-breeding cattle. Kyoto Encyclopedia of Genes and Genomes pathway analysis identified the association of DEGs with fatty acid metabolism, fatty acid degradation, peroxisome proliferator-activated receptors signaling pathway, adenosine monophosphate-activated protein kinase signaling pathway, adipocytokine signaling pathway, and the association of DE miRNAs with mitogen-activated protein kinase signaling pathway. Apolipoprotein L domain containing 1, pyruvate dehydrogenase kinase 4, and sphingosine-1-phosphate lyase 1 genes may be the key regulators of fat metabolism in grazing cattle. Finally, we found that miR-211 and miR-331-5p were negatively correlated with the elongation of very long-chain fatty acids protein 6 *(ELOVL6*), and miR-331-5p might be the new regulator involved in fat metabolism. The results indicated that *ELOVL6* participated in various functions and pathways related to fat metabolism. Meanwhile, miR-331-5p, as a new regulator, might play an essential role in this process. Our findings laid a more in-depth and systematic research foundation for the formation mechanism and characteristics of adipose tissue in grazing cattle.

## 1 Introduction

Intramuscular fat in adipose tissue plays a crucial role in influencing the taste, tenderness, and flavor of beef [1]. At the same time, the percentage of intramuscular fat is one of the indicators of carcass quality, which directly affects the value of beef [2]. Adipose tissue also plays a key role in maintaining energy homeostasis [3]. Hence, the market value of beef can be understood by understanding the deposition of adipose tissue in cattle. This understanding holds significant positive implications for the beef trade [4]. Brown adipose tissue (BAT) is a unique form of adipose tissue. Its cells contain a large number of mitochondria and consume energy by generating heat. Hence, BAT is the main part of nonshivering thermogenesis, and newborns protect themselves from the cold environment through this organization. Beige adipocytes are similar to brown adipocytes, with more small lipid droplets and mitochondria that highly express uncoupling protein-1 (*UCP1*) [5,6,7]. White adipose tissue (WAT) is the main fat storage organ in the body, which is used to store excess calories when energy intake exceeds energy expenditure [8]. Mongolian cattle is the most widely distributed and abundant breed in our country, with strong adaptability and cold tolerance. It originated in the Mongolian Plateau and is widely distributed in Inner Mongolia, Northeast China, North China, and Northwest China. Mongolian cattle are the main source of milk and meat in pastoral areas, and the famous group is produced in Ujumuqin of XilinGol. In addition, the browning of WAT, which forms beige fat, is promoted when animals are exposed to cold stress [9]. Mongolian cattle have special adipose tissue called BAT, which contains high amounts of unsaturated fatty acids, has a good taste, and hence is favored by consumers. Additionally, this brown fat tissue helps Mongolian cattle survive in harsh environments. Many factors are attributed to the production of different-colored fats, including the feeding method [10,11], season [12], and age [13]. On the contrary, mitochondrial *UCP1* plays an essential regulatory role in converting white fat into brown fat [14]. The transcription factor C/EBPs (CCAAT/enhancer-binding proteins) and the nuclear receptor peroxisome proliferator-activated receptors (PPAR) superfamily govern the differentiation of white adipocytes [15], whereas the zinc finger protein family (*KLF9* and *KLF15*) acts as both transcription activators and inhibitors [16]. Together, *KLF4* and *KROX20* (Homo sapiens early growth response 2) promote the expression of C/EBPβ, facilitating the differentiation process [17]. The primary transcriptional regulators in the brown fat differentiation process are the members of two families: PPAR γ and C/EBP [17]. In contrast, many transcriptome studies have been designed in the adipose tissue of cattle. Sheng et al. used RNA-sequencing (RNA-seq) to analyze the intramuscular, subcutaneous, and perirenal adipose tissue in cattle. They detected 110 differentially expressed genes (DEGs) in the aforementioned 3 types of adipose tissue; most of the DEGs were enriched in cellular, developmental, and metabolic processes [18]. Zhou et al. performed an RNA-seq of back fat in cattle of different ages and sexes [19]. The result showed a large number of DEGs in different age and sex groups. Protein synthesis and lipid metabolism revealed differentiation in different age groups. It was speculated that adipokines might be one of the factors leading to the development of different adipose tissues in different sexes. MicroRNAs (miRNAs) regulate the development and phenotypic differences of mammalian adipose tissue. Xu et al. reported that miRNAs played an essential regulatory role in normal fat metabolism [20]. Previous studies have reported a crucial role of miRNAs in the development of BAT. Sun et al. [21] showed that the miRNA cluster miR-193b-365 was a key regulator of brown fat development, and miR-193b induced myoblasts to differentiate into brown fat cells under adipogenic conditions. Previous target analysis of Qinchuan cattle backfat miRNAs showed that miRNA25 and miRNA26 might affect lipid and fatty acid metabolism in the adipose tissue of cattle [22]. Wang et al. used a combination of expressed sequence tags and genomic survey sequences to detect the conserved miRNAs in bovine adipose tissue [23]. The prediction of miRNA target genes showed that miR-143, miR-145, miR-2325c, and miR-2361 might regulate fat deposition differently. However, RNA-seq data for the adipose tissue of Mongolian cattle (both grazing and house breeding) are still lacking. Therefore, this study analyzed the transcriptome of mRNA and miRNA of adipose tissue of grazing and house-breeding Mongolian cattle to understand the regulatory mechanisms of fat deposition in the two different feeding types of Mongolian cattle. The study aimed to construct mRNA and miRNA transcription profiles, predict miRNA target genes, and explore the regulatory relationship between mRNA and miRNA in adipose tissue. Our findings provided theoretical data for Mongolian cattle fat deposition, which might significantly improve meat quality.

## 2 Materials and methods

### 2.1 Animals

Thirty-six-month-old third-generation crossbred Simmental × Mongolian cattle were used in this study. Three grazing cattle from ManduLatu town (Xilingol League, China) served as the test group to be compared with a control group of three housed cattle from the Baotou Meng Yi Xing Farmers’ Professional Cooperative (Baotou, China). The test-group cattle freely grazed the new pastures of the desertification steppe in Sunite Left Banner; they grazed for at least 7 h each day and received all their nutrition from foraging grasses (including Gramineae), legumes, and Asteraceae. The housed cattle were fed twice daily with free access to agricultural forages composed mainly of maize straw, green hay, sunflower seed hulls, and wheat bran. Waist fat (adipose tissue) was sampled after slaughter, and the samples were cleaned with saline, placed in lyophilization tubes, and preserved in liquid nitrogen for transcriptome analysis.

The Animal Ethics Committee of the Animal Experimentation Area at Inner Mongolia Agricultural University approved all experimental procedures, ensuring compliance with the Chinese Animal Protection Law.


**Ethical approval:** The research related to animal use has been complied with all the relevant national regulations and institutional policies for the care and use of animals and has been approved by the Animal Ethics Committee of the Animal Experimentation Area at Inner Mongolia Agricultural University.

### 2.2 RNA extraction and RNA-seq

Total RNA was extracted from the adipose tissue of six cattle using the TRIzol reagent (Invitrogen, CA, USA) following the manufacturer’s protocols. The quantity and purity of the total RNA were analyzed using the Bioanalyzer 2100 and RNA 6000 Nano LabChip Kit (Agilent, CA, USA), with RNA integrity number >7.0. Approximately 1 μg of total RNA was used to prepare a small RNA library using the TruSeq Small RNA Sample Prep Kits (Illumina, CA, USA) following the manufacturer’s protocols. We conducted single-end sequencing (36 or 50 bp) using an Illumina Hiseq 2500. The raw reads were processed using the ACGT101-miR program (LC Sciences, TX, USA) to eliminate adapter dimers, junk, low-complexity RNA families (ribosomal RNA, transfer RNA, small nuclear RNA, and small nucleolar RNA), and repeats. Next, the unique sequences with a length of 1,826 nucleotides were aligned to species-specific precursors in miRBase 21.0 through a BLAST search to identify both known and novel miRNAs. In the alignment, the deviations in length were allowed at both the 3′ and 5′-ends, besides one mismatch within the sequence. The differential expression of miRNAs was analyzed by performing a *t-*test on normalized deep-sequencing counts. The threshold for selecting DEGs was *P* < 0.05.

After purification, the poly(A)− or poly(A)+ RNA fractions were fragmented into smaller pieces using divalent cations and high temperatures. These cleaved RNA fragments were then reverse-transcribed using the mRNA-seq sample preparation kit (Illumina, CA, USA) to generate the final cDNA library following the manufacturer’s protocols. The average insert size for the paired-end libraries was 300 bp (±50 bp). We performed the paired-end sequencing on an Illumina Hiseq 4000. First, adapter contamination, low-quality bases, and undetermined base reads were removed using Cutadapt [24]. Then, the sequence quality was verified using FastQC (http://www.bioinformatics.babraham.ac.uk/projects/fastqc/). The reads were mapped to the cattle genome using Tophat2 [25] and Bowtie2 [26]. The StringTie [27] was used to assemble the mapped reads of each sample. The ASprofile software was used to perform qualitative analysis and statistics of alternative splicing events for each sample on the genetic model predicted using StringTie. Next, the transcriptomes of all cattle samples were combined to create a comprehensive transcriptome using Perl scripts. Furthermore, the expression levels of all transcripts were estimated using StringTie [27] and Ballgown [28]. The differential expression was defined using the R package Ballgown [28] as threshold *P* value < 0.05 and |log2(Fold Change)| > 1.

### 2.3 Prediction of target genes of miRNAs and functional analysis

To identify the genes targeted by the most abundant miRNAs, we used two computational target prediction algorithms, namely TargetScan5.0 [29,30,31] and Miranda3.3a [32,33,34], for detecting miRNA-binding sites. Finally, the intersection of these two software was taken as the final target gene of the differentially expressed (DE) miRNA. Subsequently, we merged the predicted data using both algorithms and calculated the overlaps. The functional analysis of the most abundant miRNAs and mRNA involved annotating Gene Ontology (GO) terms and Kyoto Encyclopedia of Genes and Genomes (KEGG) pathways. Additionally, miRNA targets were also annotated, and the significance was indicated with a *P* < 0.05. Furthermore, the correlated DEGs and DE miRNAs were used to build a co-expression network in Cytoscape (version 3.5.1) so as to explore the interactions between miRNA and mRNA in bovine adipose tissue under different feeding conditions.

## 3 Results and discussion

### 3.1 Summary of small RNA-seq analysis

**Figure 1:**
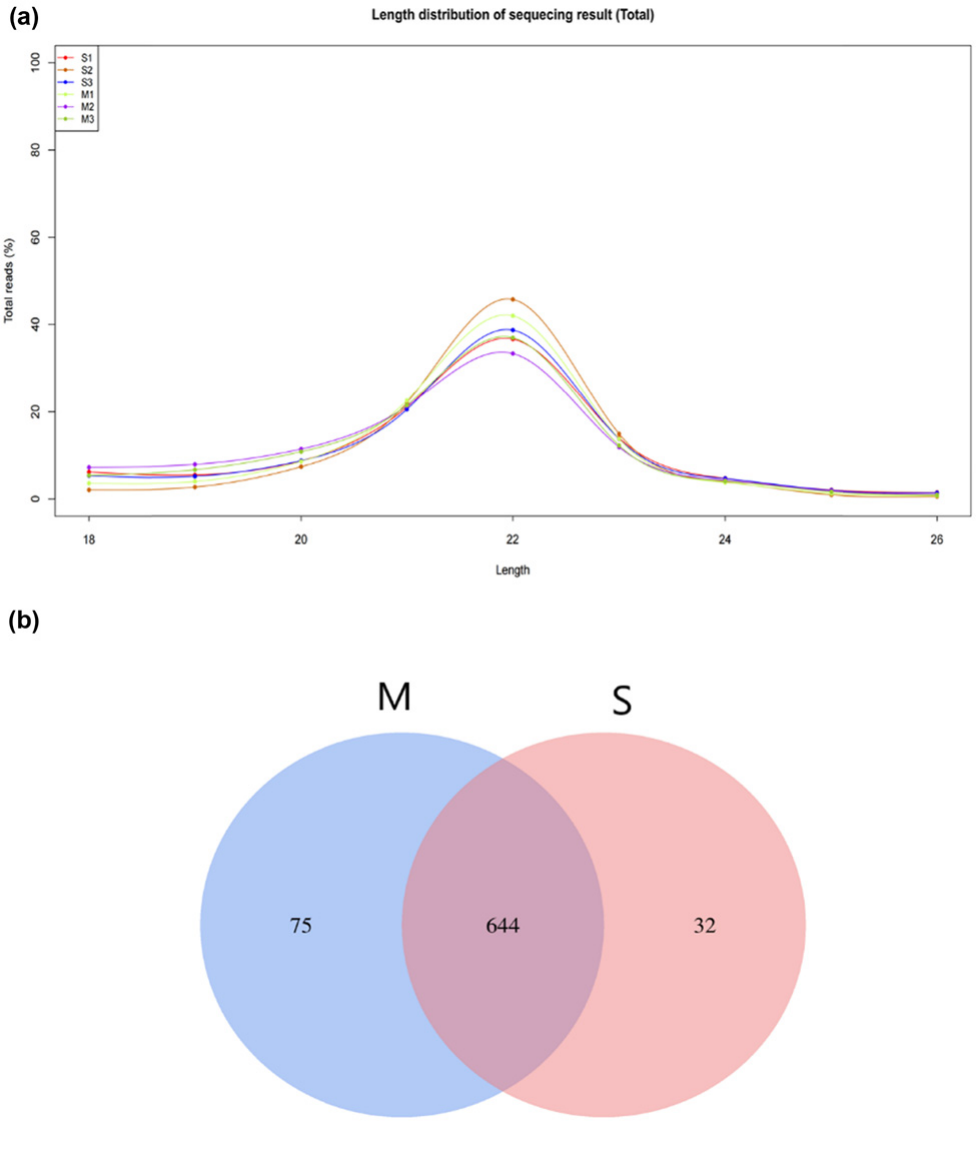
(a) Length distribution of miRNA-sequencing result. (b) Venn diagrams of detected miRNAs.

A total of 10,130,646, 17,965,472, and 14,211,676 raw reads were identified in the adipose tissue of house-breeding cattles S1, S2, and S3, respectively. Also, 17,606,103, 17,641,529, and 17,379,415 raw reads were identified in the adipose tissue of grazing cattle M1, M2, and M3, respectively. After filtering the raw reads, 6,007,209, 13,285,090, and 8,812,822 valid reads were obtained from S adipose tissue, and 12,794,057, 10,267,280, and 10,716,681 from M adipose tissue (Table 1). The length distribution statistics were analyzed for valid reads. A majority of reads were approximately 21–23 nucleotides, which conformed to the typical characteristics of Dicer enzyme cleavage (Figure 1a). Additionally, 751 miRNAs were identified in the adipose tissue of cattle fed using two different feeding methods. Furthermore, 644 miRNAs were commonly expressed in the adipose tissue of cattle fed using the two feeding methods, 75 miRNAs were uniquely expressed in M adipose tissue, and 32 miRNAs were uniquely expressed in S adipose tissue (Figure 1b). The differential expression analysis was conducted based on these results.

**Table 1:** Small RNA-seq in the adipose tissue of cattle

Sample	Raw reads	3ADT&length filter	Junk reads	Rfam	mRNA	Repeats	Valid reads
S1	10,130,646	2,942,964	21,968	1,083,272	68,041	110,776	6,007,209
S2	17,965,472	3,577,752	21,011	968,937	86,491	116,949	13,285,090
S3	14,211,676	3,718,607	27,732	1,559,192	84,616	150,970	8,812,822
M1	17,606,103	2,797,396	28,412	1,854,425	112,174	176,763	12,794,057
M2	17,641,529	4,353,113	42,532	2,815,168	145,530	291,081	10,267,280
M3	17,379,415	3,818,664	41,865	2,658,951	122,968	269,188	10,716,681

S1, S2, and S3 represent the adipose tissue of house-breeding cattle, and M1, M2, and M3 represent the adipose tissue of grazing cattle.

### 3.2 Summary of RNA-seq raw reads

A total of 44.25G of raw data were generated in six adipose tissues (Table 2). We obtained 43,274,912, 41,342,428, and 51,456,442 raw reads from S adipose tissue (S1, S2, and S3), and 45,950,434, 62,087,412, and 50,935,770 raw reads from M adipose tissue (M1, M2, and M3), respectively. Furthermore, 43.51G of valid data were obtained after filtering out low-quality reads, and on average, the valid ratio was 98%. On the contrary, the percentage of Q20 and Q30 was 99 and 94%, respectively, with GC content being 49% on average. This showed that the RNA-seq results were highly reliable.

**Table 2:** RNA-seq quality control in the adipose tissue of cattle

Sample	Raw data		Valid data		Valid ratio (reads)	Q20%	Q30%	GC content (%)
	Read	Base	Read	Base				
S1	43,274,912	6.49G	42,474,644	6.37G	98.15	99.52	94.49	49
S2	41,342,428	6.20G	40,628,566	6.09G	98.27	99.31	94.32	50.50
S3	51,456,442	7.72G	50,586,680	7.59G	98.31	99.67	95.39	50
M1	45,950,434	6.89G	45,176,702	6.78G	98.32	99.55	95.00	49
M2	62,087,412	9.31G	61,101,038	9.17G	98.41	99.61	95.16	50
M3	50,935,770	7.64G	50,051,224	7.51G	98.26	99.63	95.42	50

S1, S2, and S3 represent the adipose tissue of house-breeding cattle, and M1, M2, and M3 represent the adipose tissue of grazing cattle.

### 3.3 Differently expressed miRNA in the adipose tissue of cattle

**Figure 2:**
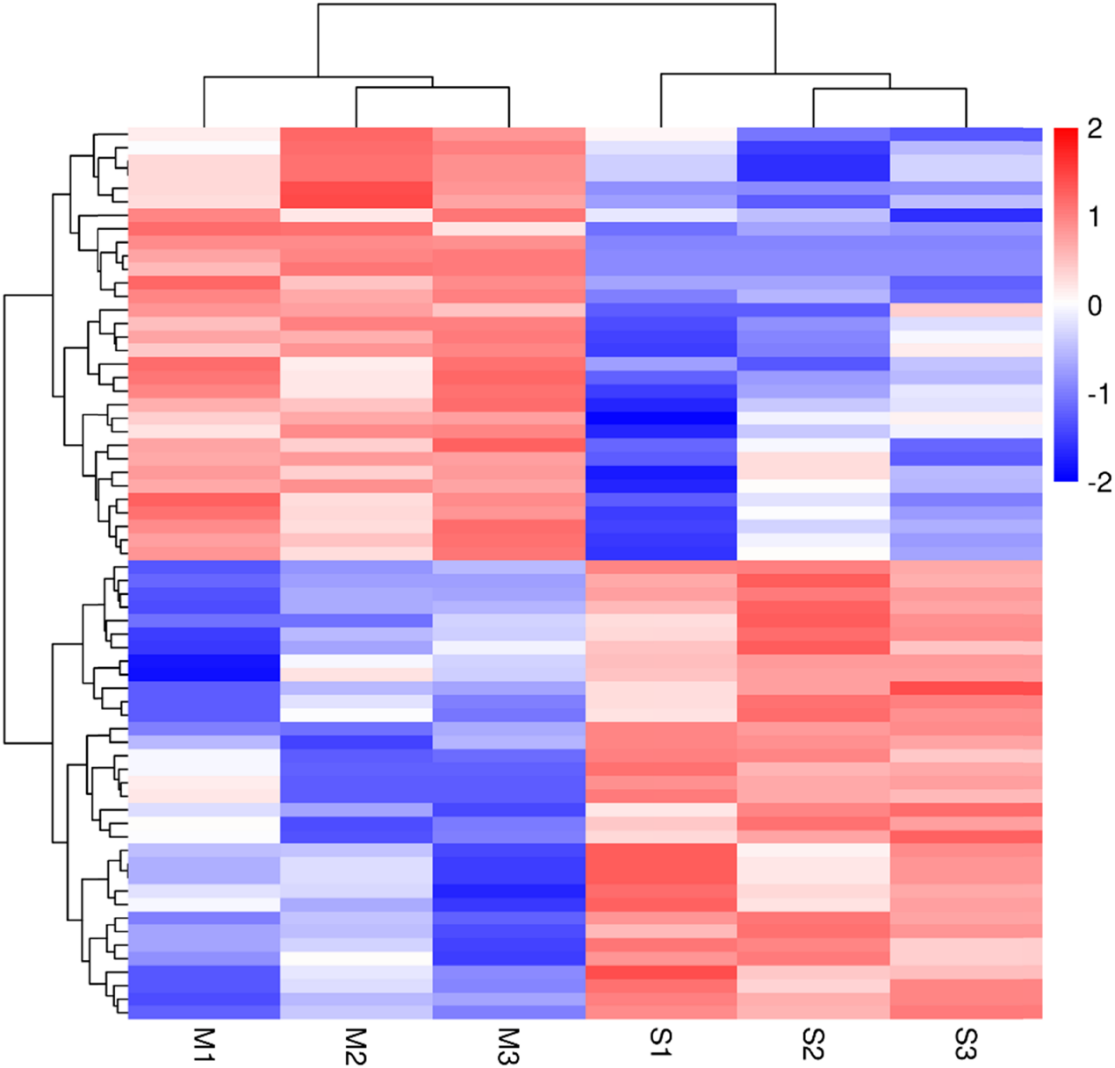
Hierarchical clustering of DE miRNAs.

We compared the adipose tissue of cattle fed by two different feeding methods using |log2(Fold Change)| ≥1 and *P* value <0.05 to identify the DE miRNAs. In the comparison group of M versus S (control group), we identified 66 DE miRNAs, including 32 upregulated miRNAs and 34 downregulated miRNAs. Among these DE miRNAs, we found 15 highly expressed miRNAs, of which 4 miRNAs showed upregulation and 11 showed downregulation (Table 3). Further analysis of these highly expressed miRNAs revealed that three of them (miR-497, miR-146a, and miR-193b) had extremely significant expression with downregulation (*P* < 0.01). The study showed that miR-497 regulated biological processes associated with triglyceride, cholesterol, and unsaturated fatty acid synthesis in cells [35]. MiR146a-5p played a key role in intramuscular fat deposition and was shown to inhibit the proliferation and differentiation of intramuscular preadipocytes [36]. MiR-193b was involved in regulating brown adipocyte differentiation [37]. Oliverio et al. found that deleting miR-193 in brown adipocytes attenuated the expression of key thermogenic genes (*CIDEA*, *UCP1*, *PGC1a*, and *PRDM16*) [38]. These findings suggested that the variably expressed miRNAs might positively affect the metabolism of adipose tissue in cattle. The heat map (Figure 2) depicted all DE miRNAs.

**Table 3:** Highly expressed DE miRNAs in the adipose tissue of cattle

miR-name	Regulation	*P* value (*t*-test)
bta-miR-497_R-1	Down	0.00138
bta-miR-146a_R-2	Down	0.00183
bta-miR-193b_R-2	Down	0.00599
bta-miR-23b-3p_R-2	Down	0.01294
bta-miR-181a_R-1	Down	0.01734
bta-let-7a-5p	Down	0.02486
bta-miR-195	Down	0.03113
bta-miR-143_1ss22GT	Up	0.03398
bta-let-7c	Down	0.03733
bta-miR-21-5p_R-2	Down	0.03740
bta-miR-451_R-2	Up	0.03803
bta-miR-204	Down	0.04093
bta-miR-214	Down	0.04116
bta-miR-30e-5p	Up	0.04794
bta-miR-27b	Up	0.04965

### 3.4 Differently expressed mRNAs in the adipose tissue of cattle

**Figure 3:**
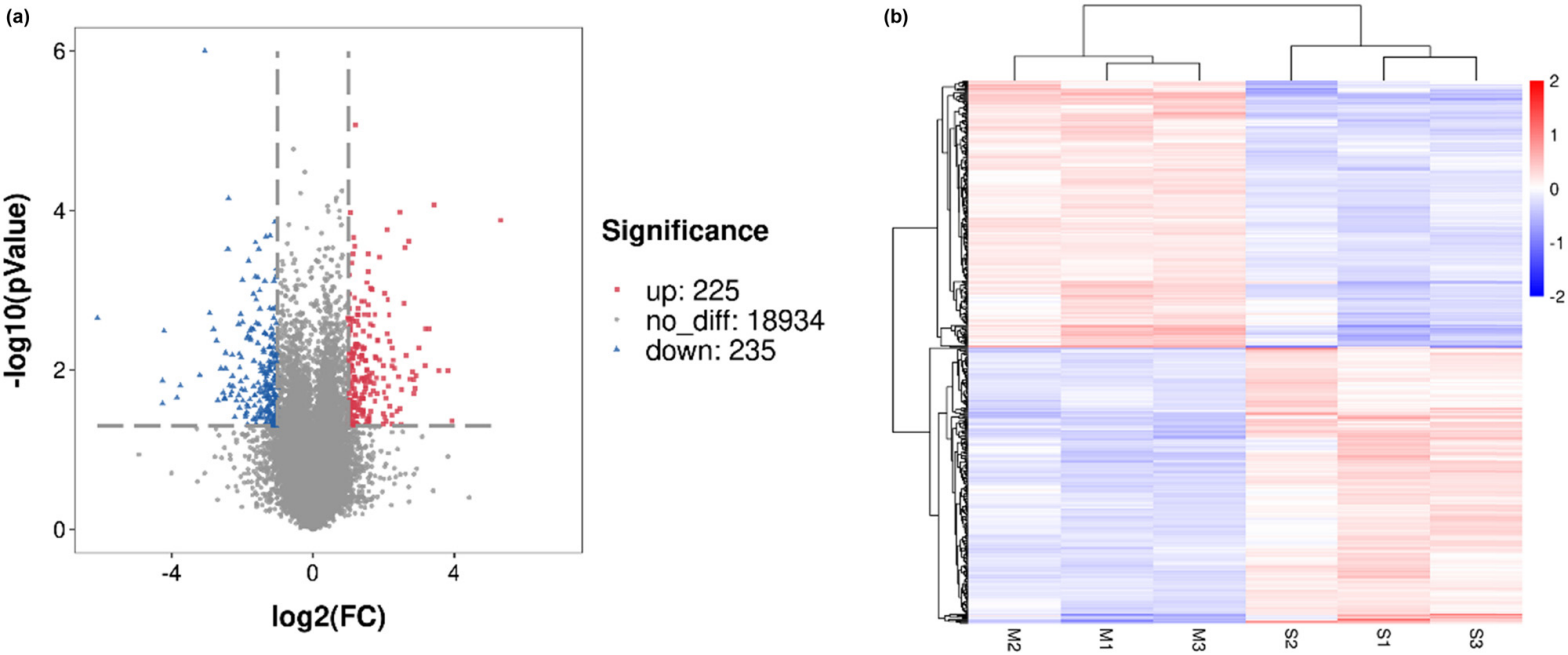
(a) Volcano plot and (b) hierarchical clustering of DEGs.

**Figure 4:**
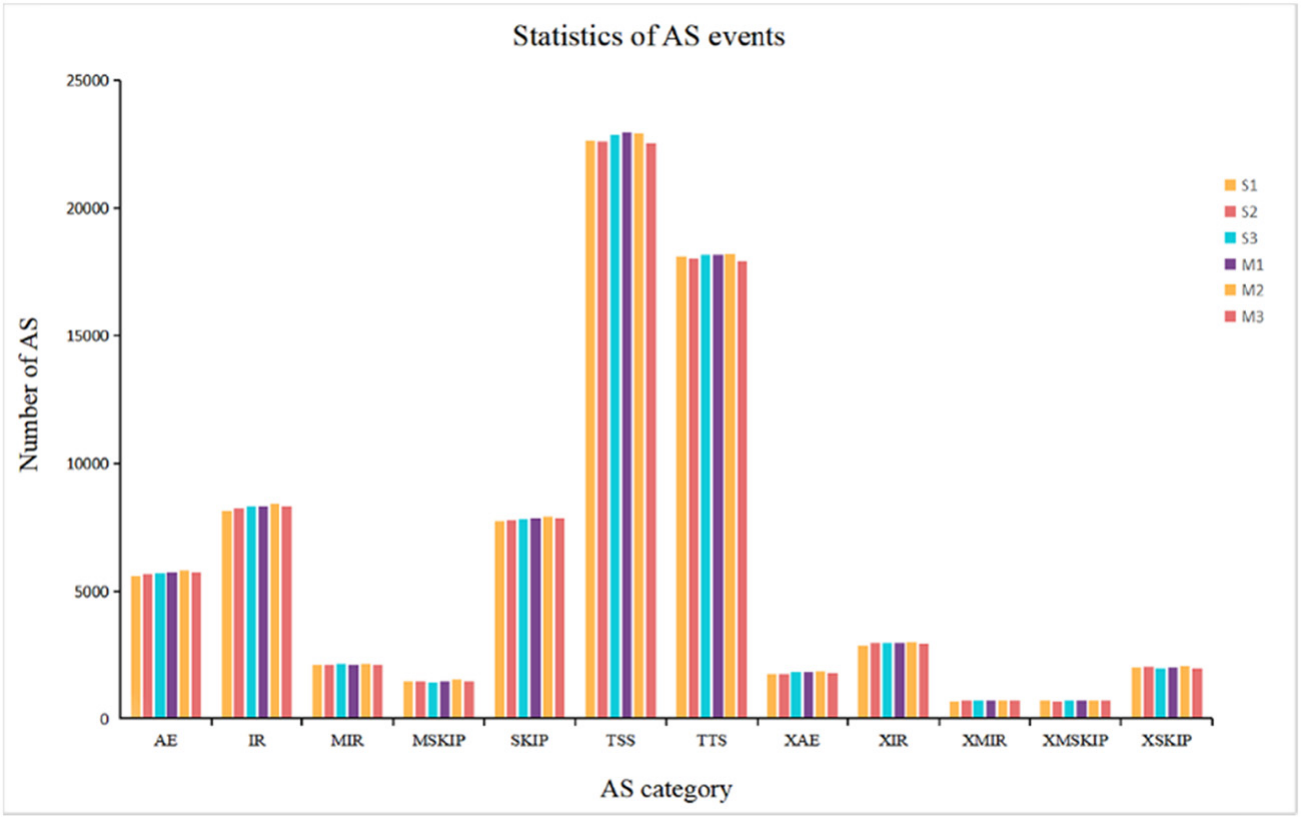
Alternative splicing analysis in cattle adipose tissue. AE, alternative exon ends; IR, retention of single introns; MIR, retention of multiple introns; MSKIP, multi-exon skipping; SKIP, exon skipping; TSS, alternative transcription start site; TTS, alternative transcription termination site; XAE, approximate AE; XIR, approximate IR; XMIR, approximate MIR; XMKIP, approximate MSKIP; XSKIP, approximate SKIP.

A total of 24,596 genes were annotated in the database. Furthermore, 460 genes were DE (*P* < 0.05) between M and S adipose tissues, of which 225 were upregulated and 235 were downregulated. The DEGs were shown in the volcano plot and heat map (Figure 3). Alternative splicing was analyzed using ASprofile software (Figure 4). The number of various types of variable shearing was extremely similar in different adipose tissues. Alternative transcription start site (TSS) occurred the most (up to 2,000), followed by alternative transcription termination site (TTS, 1,500–2,000); other types were all less than 1,000. These results showed that TSS and TTS were the main types of alternative splicing events in adipose tissue, and this was not affected by cattle breeding methods.

### 3.5 Functional analysis of DEGs

**Figure 5:**
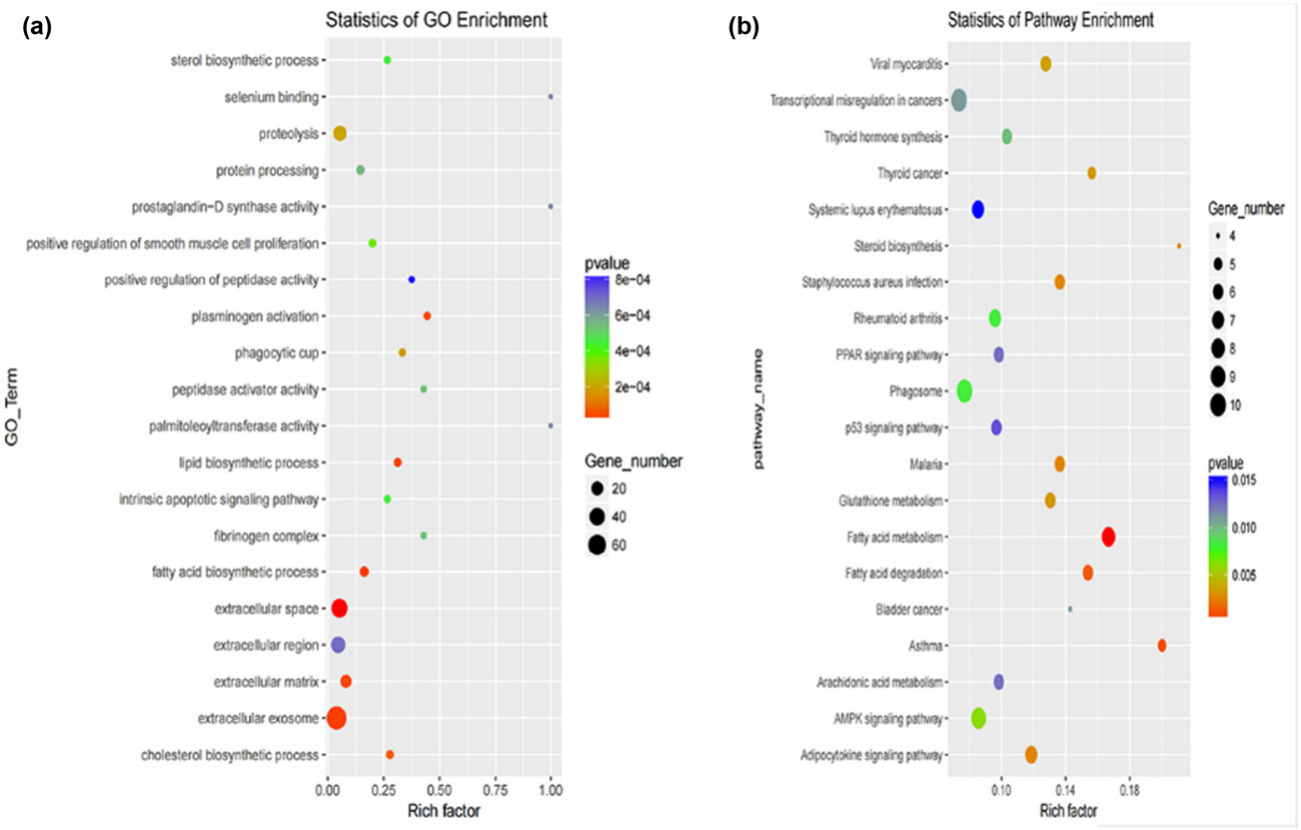
Analysis of top 20 GO terms and KEGG pathways for DEGs. (a) GO analysis of two comparison groups. (b) KEGG analysis of two comparison groups.

We analyzed the top 20 GO terms and KEGG pathways to determine the role of DEGs (Figure 5). Cellular components, biological processes, and molecular functions were determined in GO terms. We discovered that a majority of GO terms were enriched in the biological process. Among these, cellular components had the highest number of genes, particularly in the membrane-related GO terms. We identified 5,219 background genes and 141 significantly expressed genes that were enriched in these GO terms. In the two types of adipose tissue, the DEGs were significantly enriched in 39 GO terms, all of which were related to the cellular component; the extracellular area was the most. On the contrary, five DEGs were significantly enriched in lipid particles (*P* < 0.05): apoptosis-inducing mitochondrion-associated factor 2 (*AIFM2*), diacylglycerol O-acyltransferase 2 (*DGAT2*), Type 11 hydroxysteroid (17-beta) dehydrogenase (*HSD17B11*), lipase E (*LIPE*), and lanosterol synthase. *AIFM2* is a lipid-droplet-associated protein highly and specifically expressed in brown fat adipose tissue. A previous study showed that the *AIFM2* expression played an important role in thermogenesis in BAT [39]. In our study, *AIFM2* was significantly highly expressed in M adipose tissue than in S adipose tissue, and this might be one of the markers to distinguish between brown fat and white fat in cattle. As a core catalyst for triacylglycerol (TG), *DGAT2* plays an essential role in mediating the TG synthesis of fatty acids synthesized *de novo* [40,41]. The overexpression of *HSD17B11* can promote the accumulation of lipid droplets in *Caenorhabditis elegans* and HeLa cells and, at the same time, induce the accumulation of TG in HeLa cells [42]. The *LIPE* gene was found to be highly expressed in both subcutaneous and visceral fat of cattle. Furthermore, the overexpression of hormone-sensitive lipase in bovine fetal fibroblasts led to the reverse regulation of lipogenesis-related genes, thereby affecting fatty acid metabolism [43]. However, the analysis of KEGG pathways revealed that 33 of them were significantly enriched in cattle adipose tissue. Several fat-related pathways and enriched DEGs were displayed in the co-network to uncover the mechanism of fat deposition. In more specific terms, carnitine palmitoyl transferase 1a, carnitine palmitoyl transferase 1C, and *ACSBG1* (acyl-CoA synthetase bubblegum family member 1) were enriched in multiple pathways, including fatty acid metabolism, fatty acid degradation, adipocytokine signaling pathway, PPAR signaling pathway, and adenosine monophosphate-activated protein kinase (AMPK) signaling pathway. In addition, elongation of very long-chain fatty acids protein 6 *(ELOVL6*) was significantly enriched in fatty acid metabolism in our study. *ELOVL6*, a type of rate-limiting enzyme, plays an essential role in adipose tissue by catalyzing the extension of fatty acids [44]. The overexpression of *ELOVL6* showed that the ratio of C14:0 (myristic acid) and C16:0 (palmitic acid) fatty acids in adipocytes decreased, whereas the ratio of C18.0 (stearic acid) and C20:4n6 (arachidonic acid) fatty acids in adipocytes elevated [45]. The transcriptome and genomic sequencing revealed that *ELOVL6* could be a crucial candidate gene influencing beef quality [46]. In our study, *ELOVL6* was downregulated between M and S, indicating that fatty acid extension activity S was more active than M and might eventually affect beef quality.

### 3.6 Prediction and functional analysis of miRNA target gene

**Figure 6:**
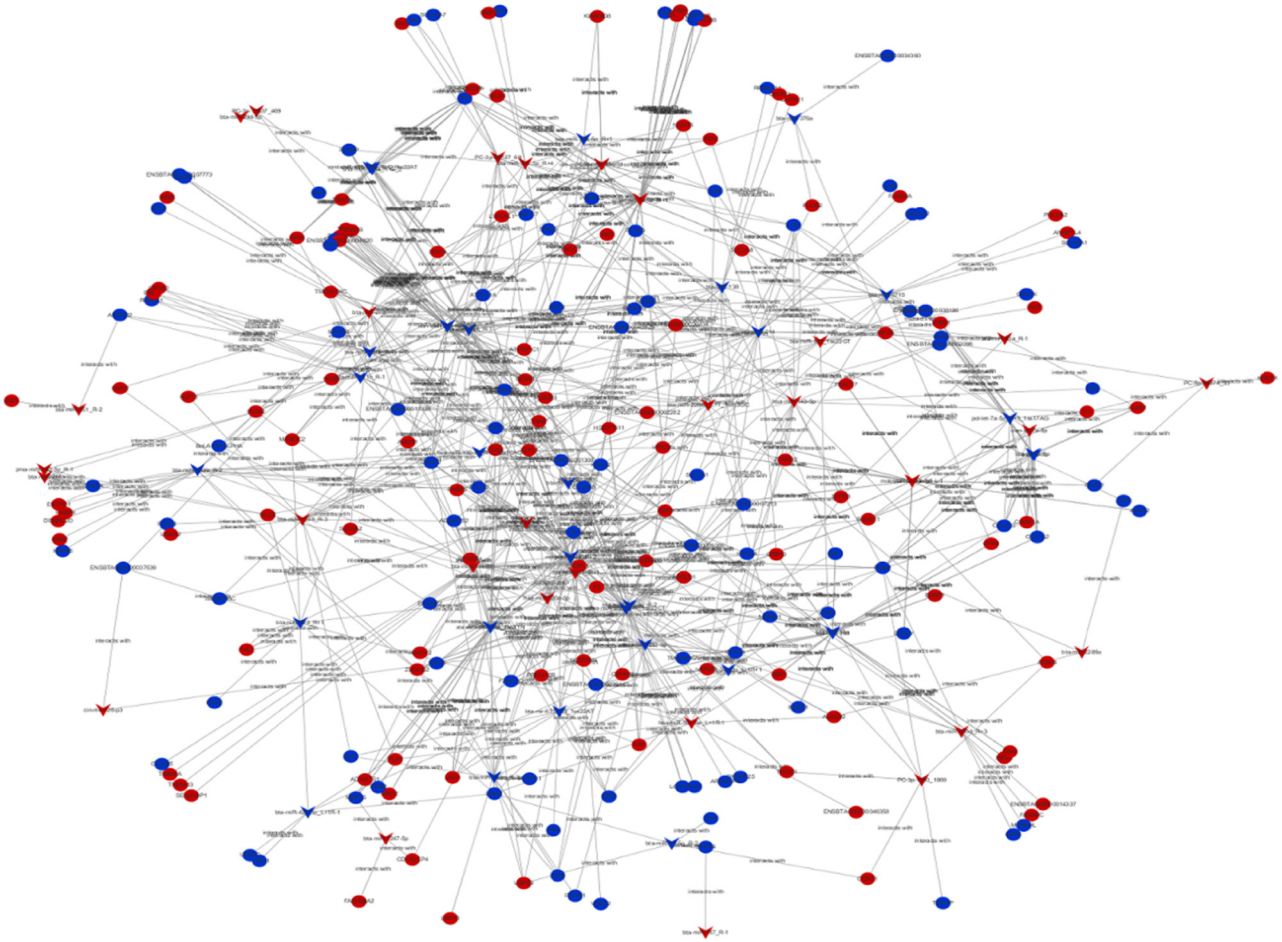
Co-expression network of differently expressed mRNA–miRNA. Circles and triangles represent mRNAs and miRNAs, respectively; red and blue represent upregulation and downregulation, respectively.

We employed TargetScan and miRanda to forecast the target genes for miRNAs. The main focus was on the pair of DEGs and DE miRNAs. A total of 66 DE miRNAs were connected to 205 DEGs and constituted 815 differently expressed mRNA–miRNA pairs (Figure 6). Furthermore, 8 miRNAs were connected to more than 20 mRNAs. Among these, upregulated miR-11986b targeted 32 DEGs and 4 targets were related to fat metabolism, including 3 upregulated apolipoprotein L domain containing1 (*APOLD1*), pyruvate dehydrogenase kinase 4 (*PDK4*), and sphingosine-1-phosphate lyase 1 (*SGPL1*) and 1 downregulated *HACD4*(3-hydroxyacyl-CoA dehydratases 4). Sosa-Madrid et al. found the involvement of *APOLD1* in lipid transport or control of adipocyte function [47]. *PDK4* is involved in glycerol production in WAT [48,49]. Bektas et al. described substantial increases in the levels of serum phospholipids, triglycerides, and cholesterol in global *SGPL1*-knockout mice [50]. Therefore, the upregulated *APOLD1*, *PDK4*, and *SGPL1* genes might be the key regulators of fat metabolism in grazing cattle. Studies have shown that *HACD1-4* catalyzes the third step of the four steps of fatty acid extension, but it has no detectable activity in the fatty acid extension pathway [51]. Although *HACD4* was DE in our study, its expression level was low. This might be due to tissue specificity [52]. At the same time, we speculated that it might also be due to the negative correlation between *HACD4* and miR-11986b, which led to reduced activity. In addition, the key enzyme that acted on fatty acids mentioned earlier, *ELOVL6*, had six DE miRNAs targeting this gene at the same time. Including miR-204 and its different types miR-204_R + 2, miR-23b and with stem rings miR-23b-3p showed downregulation, miR-211 and miR-331-5p showed upregulation. Several studies reported that miRNAs targeted ELOVL6 in different species and were related to fat deposition, such as miR-144 in duck liver [53,54], miR-125a and miR-125b in pigs intramuscular fat and gooses liver [55,56], miR-25a in mice [57,58], and gga-miR-221-5p in chicken liver [59]. Furthermore, bta-miR-204 was significantly expressed in adult bovine adipose tissue in different development stages of cattle (fetal, calf, and adult bovines); it directly targeted *ELOVL6*, thus regulating adipocyte differentiation [60]. MiR-331-5p negatively regulates phosphofructokinase mRNA [61], and phosphofructokinase deficiency leads to reduced fat storage in mice [62]. In our study, miR-331-5p was upregulated, leading to increased fat storage in grazing cattle. No study showed that miRNA-331-5p targeted *ELOVL6* to regulate fat metabolism. So, we indicated that miR-331-5p might be the new regulator for *ELOVL6* to participate in fat metabolism.

**Figure 7:**
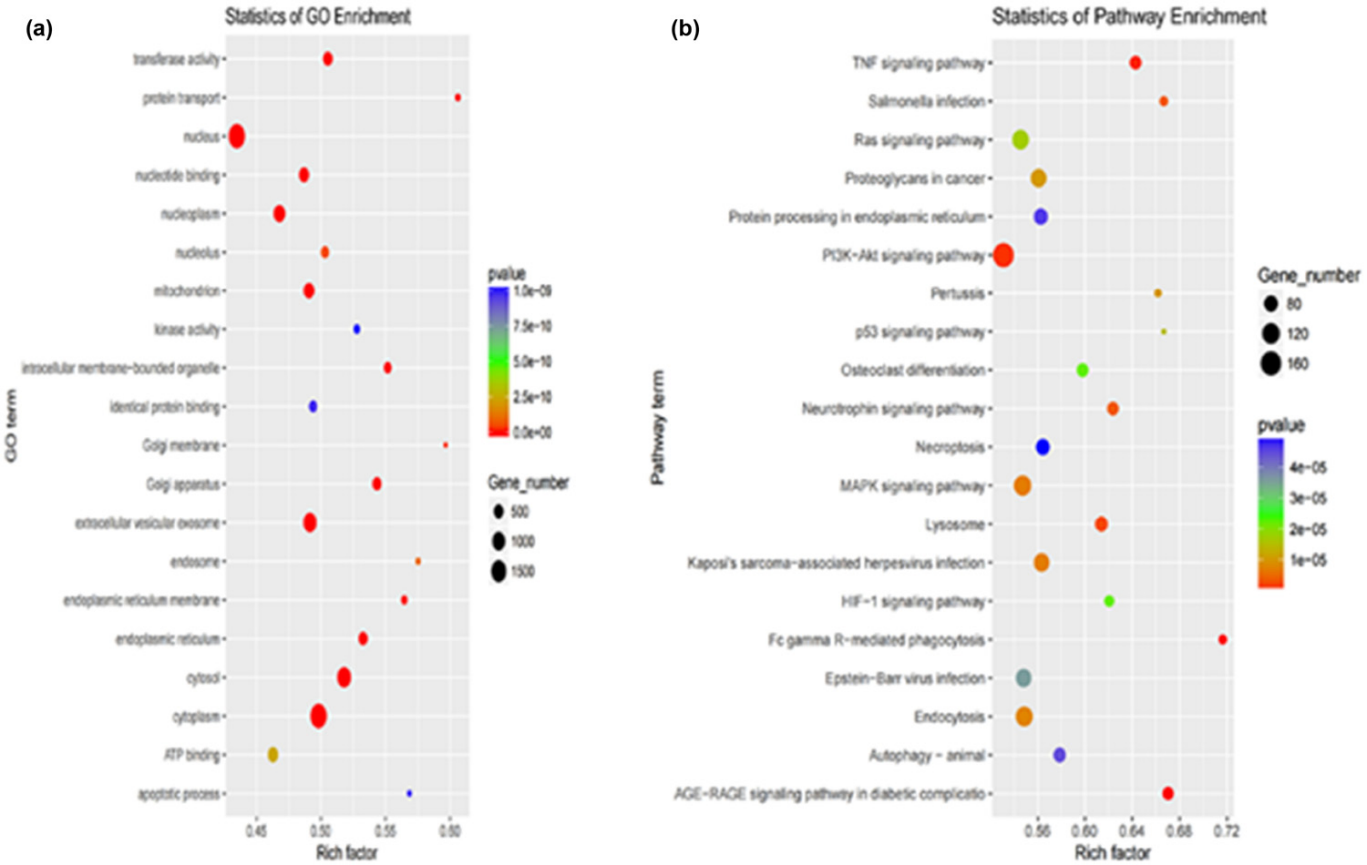
Analysis of top 20 GO terms and KEGG pathways for DE miRNAs. (a) GO analysis of two comparison groups. (b) KEGG analysis of two comparison groups.

The analysis of the top 20 GO terms and KEGG pathways is shown in Figure 7. The miRNA target genes in cattle adipose tissue were significantly enriched in 773 GO terms (*P* < 0.05); these GO terms were mainly focused on the biological process. Positive regulation of fat cell differentiation (*P* < 0.001), fatty acid metabolic process (*P* < 0.05), and long-chain fatty acid biosynthetic process (*P* < 0.05) were highly enriched in cattle adipose tissue. A significant enrichment of 144 KEGG pathways was observed in cattle adipose tissue (*P* < 0.05). The mitogen-activated protein kinase (MAPK) signaling pathway (*P* < 0.001), AMPK signaling pathway (*P* < 0.05), phospholipase D signaling pathway (*P* < 0.05), and adipocytokine signaling pathway (*P* < 0.05) were potential therapeutic pathways in both M and S adipose tissues, with the MAPK signaling pathway being particularly promising. Ji et al. identified the MAPK signaling pathway as a key signaling pathway in adipose tissue development in yak [63]. Insulin secretion controls lipid accumulation in precursor adipocytes, hence regulating adipose tissue metabolism [64]. The MAPK signaling pathway plays an essential role in insulin secretion [65], and therefore, it is related to lipid metabolism [66]. Overall, these highlighted GO terms and KEGG pathways might be important in determining the correlation between mRNAs and miRNAs and synergistically regulate fat metabolism in the adipose tissue of cattle fed by two different feeding methods.

## 4 Conclusions

The purpose of this transcriptome analysis was to reveal the molecular mechanism underlying fat metabolism in the adipose tissue of cattle using two distinct breeding methods. A total of 456 DEGs and 66 DE miRNAs were identified in cattle adipose tissue. The GO and KEGG pathway analysis showed that DEGs and miRNA target genes focused on several fat-related functions. More importantly, the predictive analysis of miRNA target genes concluded that miR-331-5p might be a new regulator targeting *ELOVL6* and was involved in fat metabolism. These findings provided a better understanding of fat metabolism in the adipose tissue of cattle fed by two different feeding methods and also basic theoretical data for improving beef quality.
